# Hernia in Children

**Published:** 1880-12

**Authors:** 


### HERNIA IN CHILDREN.

SWASEY considers this a very important matter, especially for the reason that during the early years of life, particularly the first decade, more conditions simulating hernia are found, than in later years; for instance, encysted hydrocele of the spermatic cord, congenital hydrocele, and a testicle lodged in the inguinal canal or just below the external rings, not yet having completed the descent into the scrotum.

Of 6,469 cases of hernia taken from English reports, about one-half occurred during the first five years of life, and about two-fifths of the whole number during the first year. Of Dr. Swasey's 500 cases (under twenty years of age), which include 411 of inguinal and femoral hernias, four-fifths occurred in the first five years, and two-fifths in the first year.

Femoral hernia during childhood is very rare; in the 500 cases before us only four are noted—the reason for this infrequency being that the pelvis has not reached full expansion, and the points of attachment for Poupart's ligament are comparatively near together, so that the depth of the arch formed by this ligament and the border of the bony pelvis opposite, is too shallow at the situation of the femoral opening to give lodgement for a protrusion.

In women, especially if multiparous, the iliac wings are more expanded, the crural arch deepened and muscles are less developed than in men; while in old age, muscles and tissues have become atrophied and the passage is less strongly guarded.

The connection between phimosis and inguinal hernia as before brought to notice by Kempe (*London Lancet*, 1878,) was observed to occur to greater or less extent in a great majority of instances, the obstruction to urination occasioning pain and straining during the act; but the author is not disposed to consider this a well-defined cause, as hernia is very frequent and often very unmanageable among Jews who have been circumcised.

The observations of the author fail to discover any marked heredity as a predisposing cause, which has been claimed by Sir

Astley Cooper and by Kingdom, of the London Truss Society. Dr. Swasey urges the importance of treatment at the earliest stages of discovery, during infantile life, by constant wearing of a properly adapted steel-spring truss, which should only be removed for cleansing, and with the child in the dorsal position. We may expect to produce radical cure.

The truss should pass over the opposite hip to that side on which the hernia exists, the pad should usually be egg-shaped, adapted and fitted to each individual case. The parts should be kept scrupulously clean and dry, and it is well to bathe the parts with some astringent solution such as tannic acid and alum in alcohol and water. The surface on which pressure is made should be protected by soft muslin filled with powdered starch which has a gloss which will admit of motion over the surface without chafing.—*American Journal of Obstetrics.*

